# Supplementary material for: High-resolution transcriptomics of stem and storage root vascular cambia highlight regulatory processes for xylem parenchyma differentiation in cassava
Source: BMC Genomics. 2025 Dec 9;26:1095. doi: 10.1186/s12864-025-12076-w (PMC12690950; doi:10.1186/s12864-025-12076-w)
Supplement: Supplementary file 17 — Supplementary Material 17 [file 12864_2025_12076_MOESM17_ESM.docx]

## Supporting Information

**High-resolution transcriptomics of stem and storage root vascular cambia highlight regulatory processes for xylem parenchyma differentiation in cassava**

Running title: Regulation of cassava stem and storage root xylem formation

David Rüscher^1^, Uwe Sonnewald^1^, Wolfgang Zierer^1*^

*To whom correspondence should be addressed. Tel.: +49 9131 85 25238

^1^ Friedrich-Alexander-University Erlangen-Nuremberg, Department of Biology, Division of Biochemistry, Staudtstrasse 5, 91058 Erlangen, Germany

**Supplementary Figures**


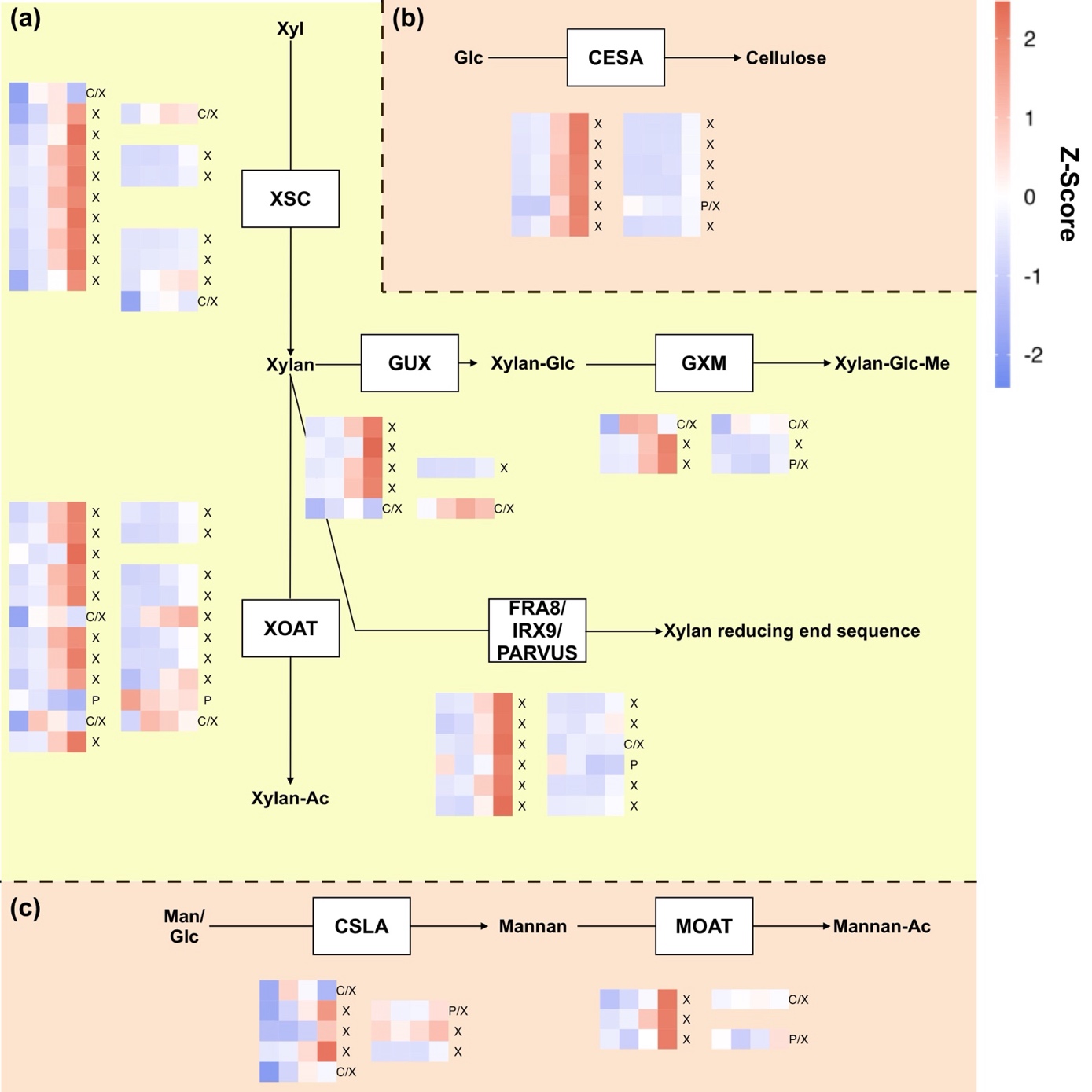


**Fig S1. Expression of genes transcribing for enzymes involved in (Hemi-) cellulose biosynthesis.**

P = Phloem cluster, P/C = Phloem/Cambium cluster, C/X = Cambium/Xylem cluster, X = Xylem cluster. Left boxes represent expression in stems, right boxes represent expression in storage roots.


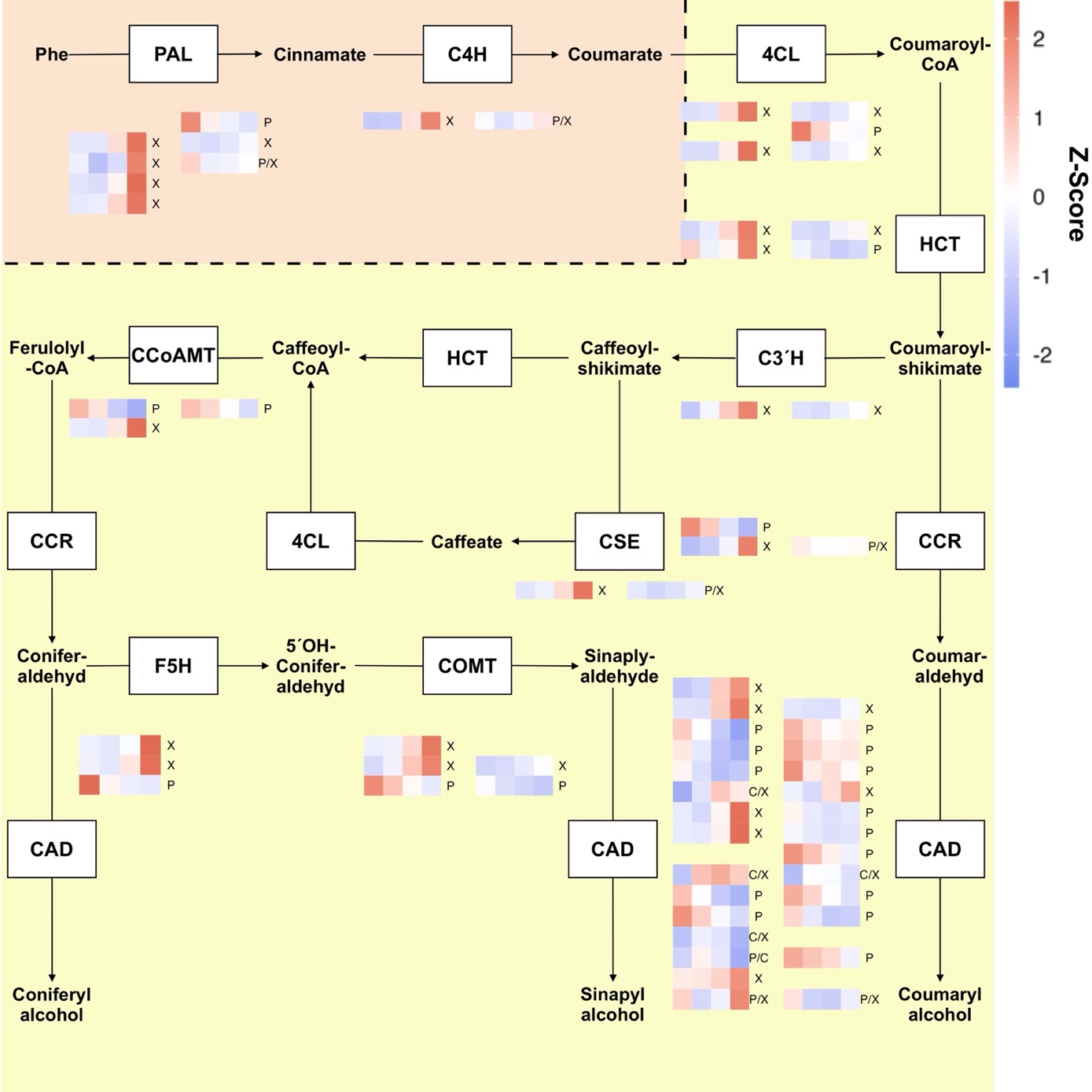


**Fig S2. Expression of genes transcribing for enzymes involved in lignin biosynthesis.**

P = Phloem cluster, P/C = Phloem/Cambium cluster, C/X = Cambium/Xylem cluster, X = Xylem cluster. Left boxes represent expression in stems, right boxes represent expression in storage roots.


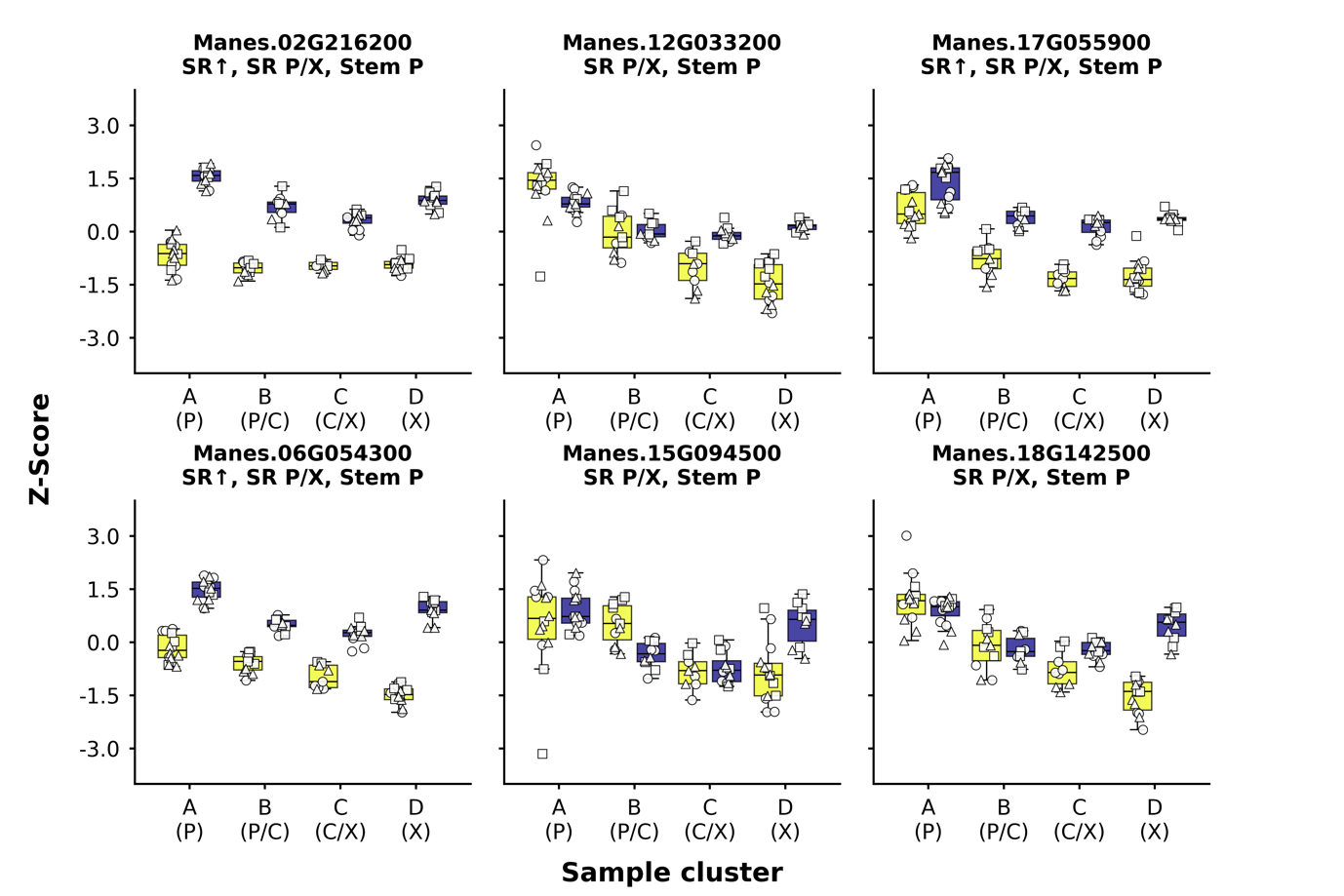


**Fig S3. Expression of *LSH* genes in stems and storage roots.**

P = Phloem cluster, P/C = Phloem/Cambium cluster, C/X = Cambium/Xylem cluster, X = Xylem cluster. Yellow boxes represent expression in stem tissue, blue boxes represent expression in storage root tissue.


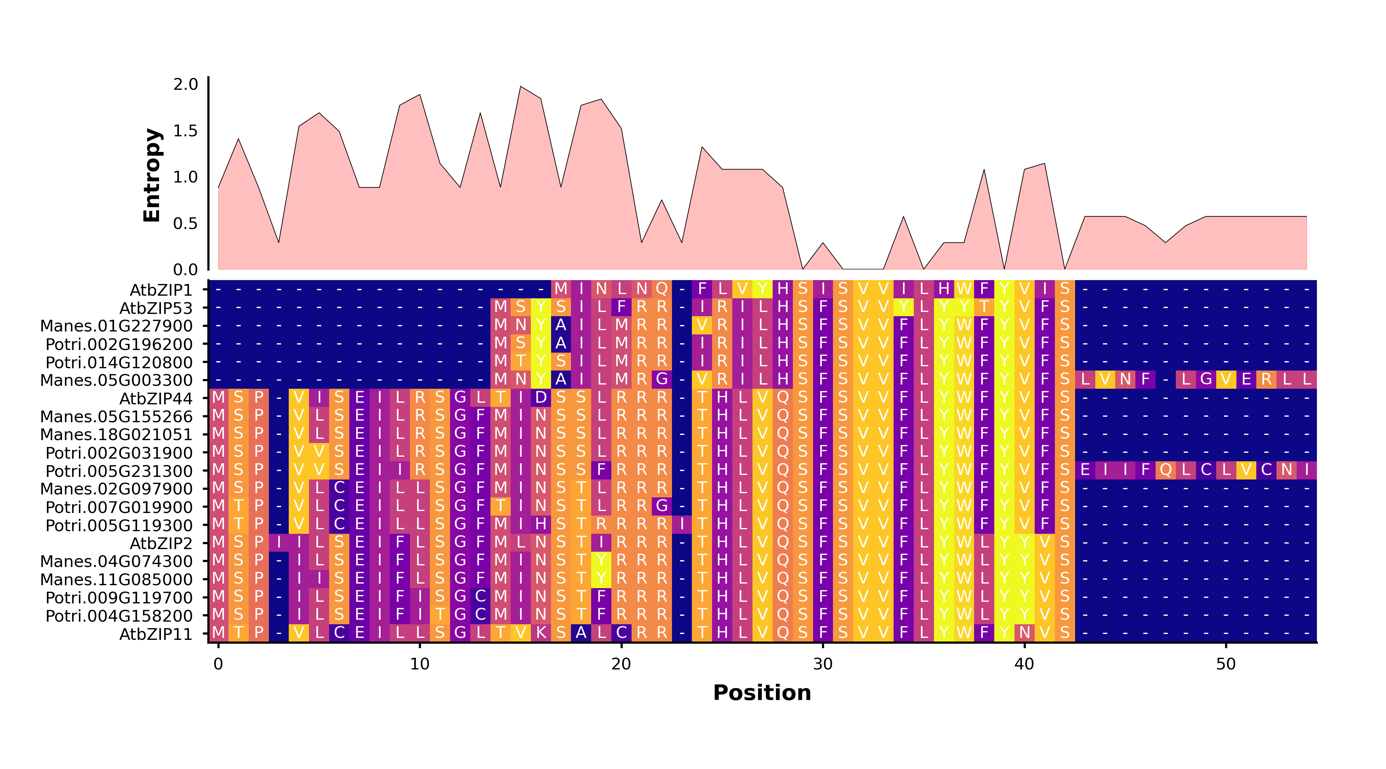


**Fig. S4. Alignment of translated SIRT-bZIP uORF from cassava, poplar, and *A. thaliana.***

**Supplementary Files**

**Supplementary File 1: Likelihood ratio test to determine differential expression results between “phloem”, “phloem/cambium”, “cambium/xylem”, and “xylem” clusters.**

CSV file containing the results of the likelihood ratio test used for testing differential expression of genes across clusters. Each row represents a gene within a tissue. Columns in order: Gene identifier, Tissue (Storage root (SR) or Stem), baseMean (global mean counts), log2FoldChange, lfcSE (standard error of the log2FoldChange), stat (test statistic), pvalue, padj (Benjamini-Hochberg adjusted pvalue).

**Supplementary File 2: Gene clustering results.**

CSV file containing gene clustering results and uniform manifold approximation projection (UMAP). For details, please see data analysis section in Material and Methods. Each row represents a gene within a tissue. Columns: index (Gene identifier), CommunityDegree (Number of edges drawn between the gene and other genes in the same community), Cluster (The cluster/community the gene is part of), Tissue (SR or Stem), UMAP1 (First UMAP projection axis), UMAP2.

**Supplementary File 3: Wald differential expression test results.**

CSV file containing the results of the WALD test used for differential expression analysis of genes across tissues while controlling for cluster. Index (Gene identifier), baseMean (global mean counts), log2FoldChange, lfcSE (standard error of the log2FoldChange), stat (test statistic), pvalue, padj (Benjamini-Hochberg adjusted pvalue).

**Supplementary File 4: GO term enrichment results on the SR Low, Stem X, and SR X intersection.**

Excel file containing GO term enrichment results for the intersection of SR Low, Stem X, and SR X. Each row describes a GO term, its name as well what Ontology it is from. Only GO terms with an adjusted p-value < 0.05 are shown. Columns: waldClusterIntersection (name of the analyzed cluster intersection), Ontology, ID (GO term), Description (name of the GO term), GeneRatio (number of genes that are part of the intersection and the GO term/ Total number of genes in the intersection), BgRatio (number of genes within the GO term in the whole genome/total number of genes in the genome with a GO term), pvalue, p.adjust (Benjamini-Hochberg adjusted pvalue), qvalue (estimated false discovery rate), gene identifier (Gene identifiers that are part of the intersection and the GO term), Count (number of genes in the intersection and GO term).

**Supplementary File 5: GO term enrichment results on gene higher expressed in storage roots.**

Excel file containing GO term enrichment results for the SR High genes. Each row describes a GO term, its name as well what Ontology it is from. Only GO terms with an adjusted p-value < 0.05 are shown. Columns: waldClusterIntersection (name of the analyzed cluster intersection), Ontology, ID (GO term), Description (name of the GO term), GeneRatio (number of genes that are part of the intersection and the GO term/ Total number of genes in the intersection), BgRatio (number of genes within the GO term in the whole genome/total number of genes in the genome with a GO term), pvalue, p.adjust (Benjamini-Hochberg adjusted pvalue), qvalue (estimated false discovery rate), gene identifier (Gene identifiers that are part of the intersection and the GO term), Count (number of genes in the intersection and GO term).

**Supplementary File 6: List of cassava BLASTP hits of important secondary cell wall and vascular cambium related genes with associated expression cluster.**

CSV file containing a list of relevant cassava orthologs selected based on BLASTP results against *A. thaliana* genes that are part of secondary cell wall biosynthesis or vascular cambium formation. Columns: Gene identifier, AT_locusName (the *A. thaliana* locus used for BLASTP), Name (trivial name of the *A. thaliana* gene), Involvement (where the gene is involved in; i.e. hemicellulose biosynthesis), plotGroup (in what heatmap the gene is plotted in), clusterIntersection (what SR and/or stem gene cluster the gene is part of), waldGroup (is the gene significantly higher or lower expressed in SR), waldClusterintersection (concatenation of clusterIntersection and waldGroup), Pattern (expression pattern as discussed in the results).

**Supplementary File 7: GO term enrichment results on the SR High, and SR PX intersection.**

Excel file containing GO term enrichment results for the intersection of SR High and SR PX. Each row describes a GO term, its name as well what Ontology it is from. Only GO terms with an adjusted p-value < 0.05 are shown. Columns: waldClusterIntersection (name of the analyzed cluster intersection), Ontology, ID (GO term), Description (name of the GO term), GeneRatio (number of genes that are part of the intersection and the GO term/ Total number of genes in the intersection), BgRatio (number of genes within the GO term in the whole genome/total number of genes in the genome with a GO term), pvalue, p.adjust (Benjamini-Hochberg adjusted pvalue), qvalue (estimated false discovery rate), gene identifier (Gene identifiers that are part of the intersection and the GO term), Count (number of genes in the intersection and GO term).

**Supplementary File 8: List of all cassava genes that could be grouped into expression clusters and their expression profile.**

Excel file containing all clustered cassava genes with pattern information and expression levels. Colors in the VST value columns show scaled expression per row across sample clusters. Columns: Gene identifier, Stem Cluster (which stem cluster the gene is part of), SR cluster (which storage root cluster the gene is part of), Wald Group (if the gene is significantly higher or lower expressed in the storage root), Pattern (what expression profile the gene is part of as discussed in the results section), VST Expression (shows mean VST values +/- sd for each sample cluster after batch correction).

**Supplementary File 9: GO term enrichment results on the SR High, and SR PX intersection.**

Excel file containing GO term enrichment results for the MYB46 pattern. Each row describes a GO term, its name as well what Ontology it is from. Only GO terms with an adjusted p-value < 0.05 are shown. Columns: Pattern (name of the analyzed expression pattern), Ontology, ID (GO term), Description (name of the GO term), GeneRatio (number of genes that are part of the expression pattern and the GO term/ Total number of genes in the expression pattern), BgRatio (number of genes within the GO term in the whole genome/total number of genes in the genome with a GO term), pvalue, p.adjust (Benjamini-Hochberg adjusted pvalue), qvalue (estimated false discovery rate), gene identifier (Gene identifiers that are part of the expression pattern and the GO term), Count (number of genes in the expression pattern and GO term).

**Supplementary File 10: GO term enrichment results on the *KNOX1* expression profile.**

Excel file containing GO term enrichment results for the *KNOX1* pattern. Each row describes a GO term, its name as well what Ontology it is from. Only GO terms with an adjusted p-value < 0.05 are shown. Columns: Pattern (name of the analyzed expression pattern), Ontology, ID (GO term), Description (name of the GO term), GeneRatio (number of genes that are part of the expression pattern and the GO term/ Total number of genes in the expression pattern), BgRatio (number of genes within the GO term in the whole genome/total number of genes in the genome with a GO term), pvalue, p.adjust (Benjamini-Hochberg adjusted pvalue), qvalue (estimated false discovery rate), gene identifier (Gene identifiers that are part of the expression pattern and the GO term), Count (number of genes in the expression pattern and GO term).

**Supplementary File 11: Transcription factor binding site enrichment results.** Excel file containing TFBS enrichment results. The file contains three sheets that contain enrichment analysis results for gene clusters, cluster intersections, and expression patterns respectively. Each row describes a transcription factor. The first column is always the cluster, intersection or pattern that was tested for enrichment. Columns: ID (Gene identifier of the transcription factor), Description (Gene identifier and transcription factor family), GeneRatio (number of genes that are part of the expression group and that contain the TFBS/ Total number of genes in the expression group), BgRatio (number of genes that contain the TFBS in the whole genome/total number of genes in the genome with any annotated TFBS), pvalue, p.adjust (Benjamini-Hochberg adjusted pvalue), qvalue (estimated false discovery rate), gene identifier (Gene identifiers that are part of the expression group and that contain the TFBS), Count (number of genes that are part of the expression group and that contain the TFBS).

**Supplementary File 12: Principal component analysis results of RNA-seq samples.**

CSV file containing sample PCA results on which sample clustering was performed (for details see Data analysis section in Material and Methods). Analysis was performed independently for SR and Stem Each row describes a gene. Columns: Sample, Plant, Tissue, Section (section id), Distance (distance from cambium in µm), Cluster (sample cluster), PC1… PC48(Principal component vector)

**Supplementary File 13: Explained variances of principal components.**

CSV file containing information about the explained variance of the PCA in Supplementary File 12. Columns: PC (name of the principal component), VarExplainedPct (per cent explained variance), CumVarExplained (cumulative per cent explained variance), Tissue.

**Supplementary File 14: UMAP projection results of RNA-seq samples.**

CSV file containing sample UMAP and clustering results used for the generation of Figure 2C. Columns: Sample, Plant, Tissue, Section (section id), Distance (distance from cambium in µm), Cluster (sample cluster), UMAP1 (first UMAP projection axis), UMAP2 (second UMAP projection axis).

**Supplementary File 15: GO term enrichment results of all generated gene lists.**

Excel file containing all GO term enrichment results. The file contains multiple sheets that contain enrichment analysis results for Wald test groups, gene clusters, cluster intersections, Wald group/gene cluster intersection, and expression patterns respectively. Each row describes a transcription factor. The first column is always the cluster, intersection or pattern that was tested for enrichment. Columns: Only GO terms with an adjusted p-value < 0.05 are shown. Columns: (name of the analyzed expression group), Ontology, ID (GO term), Description (name of the GO term), GeneRatio (number of genes that are part of the expression group and the GO term/ Total number of genes in the expression group), BgRatio (number of genes within the GO term in the whole genome/total number of genes in the genome with a GO term), pvalue, p.adjust (Benjamini-Hochberg adjusted pvalue), qvalue (estimated false discovery rate), gene identifier (Gene identifiers that are part of the expression group and the GO term), Count (number of genes in the expression group and GO term).

**Supplementary File 16: List of cassava transcription factors and their expression profile.**

CSV file containing expression clusters of all cassava transcription factors. Columns: Gene identifier, Family (what transcription factor family the gene is part of), Cluster (what Wald group/gene cluster the gene is part of), Pattern (what expression pattern the gene exhibits as described in the results).
